# Developing a Mortality Risk Score in Intensive Care Units of Referral Hospitals in Bahir Dar City, Ethiopia: A Prognostic Study

**DOI:** 10.1002/hsr2.72342

**Published:** 2026-04-14

**Authors:** Yihun Miskir Wubie, Asiya Mohammed Abdu, Zelalem Alamrew Anteneh

**Affiliations:** ^1^ Department of Emergency and Critical Care Nursing, College of Medicine and Health Sciences Bahir Dar University Bahir Dar Ethiopia; ^2^ Department of Epidemiology and Biostatistics, College of Medicine and Health Sciences Bahir Dar University Bahir Dar Ethiopia

**Keywords:** Bahir Dar city, intensive care unit, mortality, referral hospitals, risk score

## Abstract

**Objective:**

The aim of this study was to develop a mortality risk score in the intensive care units of referral hospitals in Bahir Dar City.

**Methods:**

The study included 852 participants who were admitted from January 1, 2019 to December 31, 2021. We used EpiData version 3.1 for data entry and R‐software for analysis. The mortality rate among participants was 35.9%. Multivariable logistic regression was employed to identify the independent prognostic determinants. Using beta‐coefficients, we developed and validated a prognostic model. Then a mortality risk score was determined based on the value of each prognostic determinant variable.

**Results:**

Age, sex, health insurance user status, respiratory rate, temperature, mean arterial pressure, Glasgow Coma Scale, WBC count, sepsis, ARDS, organ‐insufficiency, mechanical ventilation, and vasopressor were independent prognostic determinants. Based on the prognostic determinants, we developed an easily applicable mortality risk score model. The model had a discrimination performance of AUC 0.90 (95% confidence interval of 0.88–0.92) and a calibration *p* value of 0.69.

**Conclusion:**

The prognostic determinants identified in this study are easily accessible and easy to capture in routine clinical settings. As a result, the developed model has the potential to be effectively applied in low‐income countries where resources may be limited.

**Implication for Clinical Practice:**

The model can help healthcare providers in low‐income settings to identify high‐risk patients and develop appropriate interventions to improve patient outcomes.

## 1 Introduction

Since its establishment in the 1950s [1], the intensive care unit (ICU) has become an integral part of healthcare systems worldwide. In Ethiopia, however, the introduction of ICU as a separate unit of care is a relatively new experience, started in 1993 [2]. Despite this let introduction, the expansion of ICUs in the country has been significant. The number of ICUs is steadily increasing in both government and private institutions across the country. Currently, at least 53 hospitals are equipped with either specialized or comprehensive ICUs, offering a total of 340 beds to serve a population of over 100 million [2, 3].

Patients admitted to ICU are typically in critical condition and are initially considered to have a high probability of survival [4]. However, continued aggressive treatment may not always be beneficial [5]. Merely prolonging life without meaningful recovery presents ethical and clinical challenges in ICU care and significantly increases healthcare costs [6, 7, 8]. When deciding which patients to treat, and to what extent (whether to limitation or withdrawal treatment), ICU clinicians must therefore balance technological capabilities with the wishes and expectations of patients, their families, and the healthcare teams [6, 9]. Early identification of patients who are more likely to survive enables both patients and providers to make informed decisions about care options [5, 7, 10, 11].

To support these decisions, mortality risk prediction scores or models are needed. Various such risk models have been developed and validated in high‐income countries, particularly western ICUs [12, 13, 14, 15]. These models are expected to be applicable globally [16]. However, the models are often complex, resource‐intensive, and not well‐suited to low‐resource settings [17]. Prognostication in low‐resourced ICU settings is particularly important, as many diagnostic tests used in developed settings are not readily available. Moreover, a patient's prognosis is highly influenced by the level of care that can be provided in a given context [8]. Therefore, using the best available data to guide resource allocation is not only clinically appropriate but also ethically imperative [18]. Notably, several mortality risk scores have been developed specifically for low‐income countries, most of which rely primarily on vital signs. Examples include the Rwanda mortality probability model (R‐MPM) [17], Uganda vitals [19], Tanzania vitals [20], and tropical intensive care risk score [21]. However, these models may be overly simplified and may not fully capture the complexity of critical illness. To our knowledge, no mortality risk score has yet been developed specifically for the Ethiopian ICU setting. Therefore, in this study, we aimed to develop a mortality risk prediction model in ICUs of referral hospitals in Bahir Dar city, northwest, Ethiopia.

## 2 Methods and Materials

### 2.1 Study Setting and Period

This retrospective study was conducted from January 1, 2019 to December 31, 2021, at two referral hospitals in northwest Ethiopia: Tibebe Gion Specialized Hospital (TGSH) and Felege Hiwot Comprehensive Specialized Hospital (FHCSH).

### 2.2 Study Population

We retrieved registration charts of ICU patients who were 15 years and older from March 1 to May 15, 2022. Exclusion criteria were applied to ensure data quality and the validity of the study findings. For variables with < 10% missing data, a rule‐of‐thumb approach was followed, and multiple imputation was used to handle the missing values. However, the variable bilirubin and blood urea nitrogen were excluded from the analysis due to an excessive proportion of missing data. Additionally, patients were excluded if their medical records were unavailable, if they were dead on arrival, or if they were discharged or admitted for < 24 h solely for routine postoperative monitoring.

### 2.3 Sample Size and Sampling Procedure

The sample size was determined using the rule of thumb (*n* = 10*K*/*p*) [22], which recommends at least 10 events per candidate variable. Hence, based on the literature review, around 32 candidate variables were identified. Using an ICU mortality rate of 38.7% from a comparable setting (Gonder referral hospital) [23], the required sample size was calculated as 32*10/0.387 = 827. To account for incomplete charts, we added 10% to the sample size, yielding a final sample size of 910 patients. Samples were then selected proportionally from each hospital. During the study period, 1238 and 1855 cases were admitted to the ICUs of TGSH and FHCSH, respectively. Accordingly, 364 cases from TGSH and 546 cases from FHCSH were selected using a systematic random sampling method with a k‐interval of 3 in both hospitals.

### 2.4 Study Variables

ICU mortality was considered the dependent variable, while a range of nonclinical and clinical factors were included as independent variables. Nonclinical variables comprised age, sex, health insurance status, source of referral, patient location prior to ICU admission, and pre‐ICU hospital stay. Clinical variables included initial vital signs recorded at ICU admission (mean arterial pressure, heart rate, respiratory rate, temperature, and oxygen saturation), mental status at admission, laboratory findings, and the primary admission diagnosis. Laboratory variables consisted of baseline complete blood count, serum electrolytes (sodium and potassium), creatinine, blood urea nitrogen, and bilirubin. Treatment‐related variables assessed within the first 24 h after ICU admission included the use of mechanical ventilation, vasopressors, and surgical interventions performed prior to or during the ICU stay. Complications occurring at or within the first 24 h of admission such as sepsis, acute respiratory distress syndrome, organ failure, and suspected or confirmed infection were also considered as independent variables. These variables were selected based on their clinical relevance and availability in the medical records.

Although predictor variables were collected at ICU admission or within the first 24 h of ICU stay, the model was designed to predict overall ICU mortality. Therefore, the outcome of interest included deaths occurring at any time during the ICU stay, which could occur days or weeks after admission.

### 2.5 Data Management

A pretested chart review checklist was used to extract data available within 24 h of admission (i.e. baseline laboratory‐based data ordered at admission, vital signs and mental status at admission and intervention and complication variables measured within 24 h of admission). We developed the extraction tool from various published literature [12, 13, 18, 21, 24, 25]. To ensure data quality, data collectors and supervisors received training on data extraction from charts before data collection. Furthermore, errors were checked daily, and check codes were employed during data entry. Double data entry and validation was performed using EpiData Version 3.1 and then exported to R software for statistical analysis.

We conducted a binary logistic regression analysis to identify candidate predictors of mortality among ICU patients. Variables with a *p* value of < 0.25) in the univariable model were recruited for the multivariable regression. Using a backward stepwise regression method, we determined independent predictors of mortality. During the stepwise procedure, variables were removed one at a time based on a removal criterion of *p* > 0.05 in the multivariable model. This process continued until all remaining variables were statistically significant at the 5% level.

During this stepwise procedure, the white blood cell (WBC) count showed borderline statistical significance (*p* = 0.05). However, retaining this variable slightly improved the overall model fit. In addition, given its well‐established clinical relevance as an indicator of systemic inflammation and infection in critically ill patients, we considered it appropriate to retain it in the final model. It should be noted that this does not imply that WBC count was prespecified a priori for forced inclusion in the model.

The area under the receiver operating characteristic curve (AUC, or C‐statistic) was used to assess model discrimination, while the Hosmer–Lemeshow statistic and *p* value were calculated to assess calibration. Additionally, the model was internally validated using 1000 random bootstrap samples to ensure its robustness. The AUC with its 95% confidence interval (CI) was obtained, and the sensitivity and specificity were calculated based on the optimal cut‐off probability of poor prognosis (ICU mortality). Furthermore, the calibration (Hosmer–Lemeshow C‐Statistic) and accuracy of the model (Brier score) were determined. To evaluate the clinical and public health impact of the model, a decision curve analysis (DCA) was performed and illustrated in a plot of a decision curve.

### 2.6 Ethical Consideration

Approval was obtained from the Ethical Review Committee of the College of Medical and Health Science, Bahir Dar University with a Protocol Number 309/2021. Permission letter was obtained from the respective hospitals. Data are kept confidential using codes. In addition, all methods were performed in accordance with an ethical guideline for studies involving human participants. However, informed consent was not taken as it was not applicable for a retrospective study, and this was also approved by the committee.

## 3 Results

### 3.1 Nonclinical Baseline Characteristics of Participants

Out of the total 910 screened charts, 852 charts were included giving a response rate of 93%. Male participants were higher 451 (53%) than females 401 (47%). The median age of participants was 40 years with an IQR (27, 60). Majority of the participants were not community health insurance users. Another hospital was the main source of referral, and most patients were admitted to ICU from the emergency department. The median ICU stay was 4 days with an IQR (3, 7) (Table 1).

**Table 1:** Nonclinical baseline characteristics of participants in Bahir Dar city referral hospitals, 2019–2021.

Variable	Category	Total *n* (%)	Survivor (%)	Nonsurvivor (%)
Sex	Male	451 (52.9)	272 (60.3)	179 (39.7)
	Female	401 (47.1)	274 (68.3)	127 (31.7)
Community health insurance users	Yes	320 (37.6)	221 (69.1)	99 (30.9)
	No	532 (62.4)	325 (61.1)	207 (38.9)
Sources of patients	Self/home	96 (11.3)	69 (71.9)	27 (28.1)
	Another hospital	414 (48.6)	271 (65.5)	143 (34.5)
	Health center	104 (12.2)	66 (63.5)	38 (36.5)
	Private clinic/hospital	160 (18.8)	94 (58.8)	66 (41.3)
	Accident scene	78 (9.2)	46 (59.0)	32 (41.0)
Pre‐ICU location	Emergency room	559 (65.6)	354 (63.3)	205 (36.7)
	Ward	217 (25.5)	143 (65.9)	74 (34.1)
	Recovery room	68 (8.0)	47 (69.1)	21 (30.9)
	Directly from another facility	8 (0.9)	2 (25.0)	6 (75.0)
Pre‐ICU stay in days	Below 1 day	378 (44.4)	234 (61.9)	144 (38.1)
	≥ 1 day	474 (55.6)	312 (65.8)	162 (34.2)

### 3.2 Vital Signs and Mental Status at Admission

Most (68.5%) of the patients had normal mean arterial blood pressure and the median MAP was 83.7 mmHg with an IQR of 70–98 mmHg. The mean respiratory rate (RR) and pulse rate (PR) were 25.6 ± 7 breaths/min, and 107 ± 23.8 beats/min, respectively. The median hourly urine output was 60.4 mL with an IQR (41.7, 77). Regarding mental status, around 30% of participants were comatose at admission (Table 2).

**Table 2:** Vital signs and mental status (at admission) of participants in Bahir Dar city referral hospitals, 2019–2021.

Variable	Category	Number	Percent
Mean arterial pressure (mmHg)	70–110	584	68.5
	< 70/> 110	268	31.5
Respiratory rate (breaths/min)	12–24	395	46.4
	< 12/> 24	457	53.6
Pulse rate (beats/min)	60–100	329	38.6
	< 60/> 100	523	61.4
Temperature (°C)	36.5–38.5	376	44.1
	< 36.5/> 38.5	476	55.9
SpO_2_ (%)	≥ 90	465	54.6
	< 90	387	45.4
Glasgow Coma Scale (GCS)	13–15	428	50.2
	9–12	171	20.1
	≤ 8	253	29.7

### 3.3 Baseline Complete Blood Count and Blood Chemistry

A total of 65.0%, 77.6%, and 62.9% of patients had normal values of serum sodium (Na^+^), potassium (K^+^), and creatinine, respectively. Similarly, 74.1%, 72.3%, and 62.0% of participants had normal counts of platelets, hemoglobin (Hgb), and WBC, respectively. Referenced normal values were Na^+^ (135–145 mmol/L), K^+^ (3.5–5.5 mmol/L), creatinine (0.1–1.2 mmol/L), Hgb (11–16 g/dL), WBC (4000–12,000 cells/µL), and platelets (150,000–450,000 cells/µL).

### 3.4 Interventions and Complications at or After ICU Admission (Within 24 h)

Among the 852 participants, about 42% had one or more chronic comorbidities. At or within 24 h after ICU admission, 217 patients (25.5%) developed sepsis, and 233(27.3%) developed acute respiratory distress syndrome. In total, 439 patients (51.5%) had a suspected or confirmed infection, and 45% experienced severe organ system insufficiency. Regarding interventions, 42.7% of patients were on mechanical ventilation and 16.0% required vasopressors within 24 h of ICU admission. Additionally, 169 patients (19.8%) underwent surgery, of whom 109 (64.5%) underwent emergency surgery.

### 3.5 Mortality and Its Predictors in the Intensive Care Unit

Of the 852 cases, 306 (35.9%, CI: 32.7–39.1) died during the observation period. To identify candidate predictors of mortality among ICU patients, we conducted a univariable regression. Accordingly, about 24 variables were screened (*p* < 0.25) for multivariable regression. Of those fitted variables, age, sex, health insurance user status, respiratory rate, temperature, mean arterial pressure, Glasgow Coma Scale, WBC count, sepsis, ARDS, organ‐insufficiency, mechanical ventilation, and vasopressor remained in the final model. The beta coefficients obtained after running the multivariable analysis and after 1000 times bootstrapping are depicted in the table below. As a matter of fact, the two beta coefficients are similar (Table 3).

**Table 3:** Multivariable analyses of participants in Bahir Dar city referral hospitals, 2019–2021.

Prognostic determinants	Multivariable analysis		
	*β* coefficient (95% CI)	*p*	AOR (95% CI)
Age	0.02 (0.01, 0.03)	< 0.01	1.02 (1.01, 1.03)
Sex (male)	0.42 (0.01, 0.83)	0.04	1.52 (1.01, 2.29)
Community health insurance (no)	0.52 (0.09, 0.97)	0.02	1.68 (1.08, 2.64)
Respiratory rate (breaths/min)	0.04 (0.01, 0.07)	0.01	1.04 (1.01, 1.07)
Temperature (°C)	−0.27 (−0.43, −0.12)	< 0.01	0.76 (0.65, 0.89)
Mean arterial pressure (mmHg)	−0.02 (−0.03, −0.01)	< 0.01	0.98 (0.97, 0.99)
Glasgow Coma Scale	−0.15 (−0.21, −0.10)	< 0.01	0.86 (0.81, 0.90)
White blood cell count (1000 cells/µL)	0.04 (0.00, 0.08)	0.05	1.04 (1.00, 1.08)
Sepsis (yes)	0.56 (0.01, 1.11)	0.04	1.75 (1.01, 3.03)
Acute respiratory distress (yes)	1.06 (0.58, 1.55)	< 0.01	2.89 (1.78, 4.71)
Organ insufficiency (yes)	1.07 (0.63, 1.51)	< 0.01	2.92 (1.88, 4.53)
Mechanical ventilator (yes)	0.79 (0.34, 1.25)	< 0.01	2.20 (1.40, 3.49)
Vasopressor (yes)	1.54 (0.94, 2.17)	< 0.01	4.66 (2.56, 8.76)

A mortality prediction model was developed based on the reduced logistic regression model. This model, using an optimal threshold determined by the Youden Index (0.41), achieved an accuracy of 83.5% (95% CI: 80.9%–86.0%), with a sensitivity of 77.8%, specificity of 86.8%, positive predictive value of 76.8%, and negative predictive value of 87.5%.

**Figure 1:**
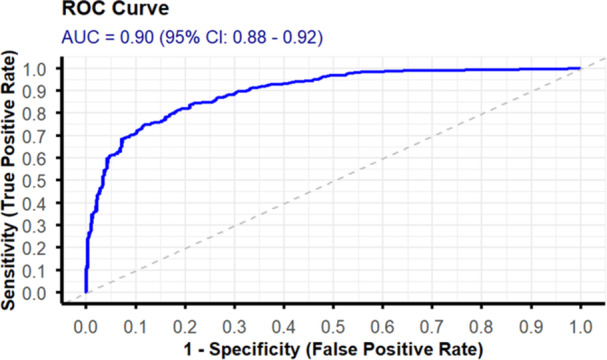
The receiver operating curve (ROC) with an area under the curve (AUC) of the original model.

The discriminatory ability of the model was evaluated using the area under the receiver operating characteristic (ROC) curve (AUC). The model demonstrated an AUC of 0.90%, with a 95% CI ranging from 0.88% to 0.92%. This AUC reflects the model's discriminatory ability to distinguish between patients who experienced the outcome and those who did not (Figure 1).

**Figure 2:**
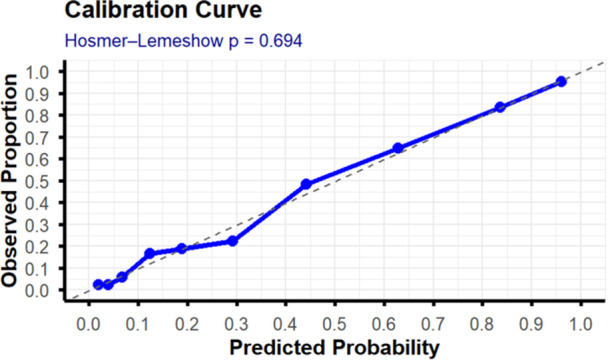
Calibration plot of the original model.

The model's goodness‐of‐fit was tested using a fitness test, which produced a *p* value of 0.69, indicating no significant difference between observed and predicted outcomes. Additionally, the calibration curve aligned nearly to the 45° reference line. This suggests that the model's predicted probabilities were well‐calibrated and aligned with actual outcomes (Figure 2).

To optimize the model and prevent overfitting, internal validation was performed using a bootstrapping technique implemented via the mrs package. After 1000 bootstrap samples with replacement used, the optimism corrected AUC was 0.88, and the optimism coefficient was estimated at 0.03.

**Figure 3:**
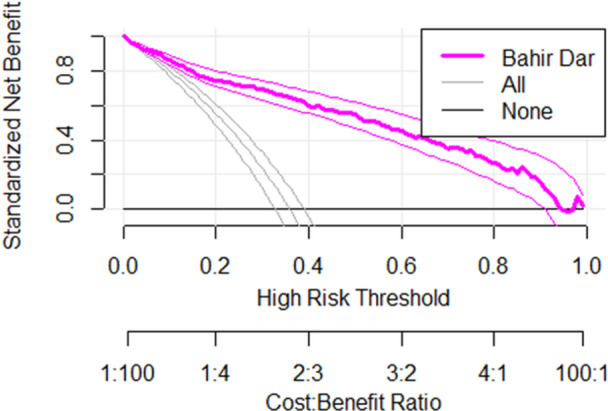
Decision curve of the validated model.

To assess the clinical utility of the model, DCA was conducted. As illustrated in the figure below, the decision curve outperforms the default strategies of treating all patients or treating none across the entire range of threshold probabilities. This implies that the model provides a net clinical benefit and can facilitate decision making (Figure 3).

### 3.6 Risk Classification and Nomogam Using a Simplified Risk Score

**Figure 4:**
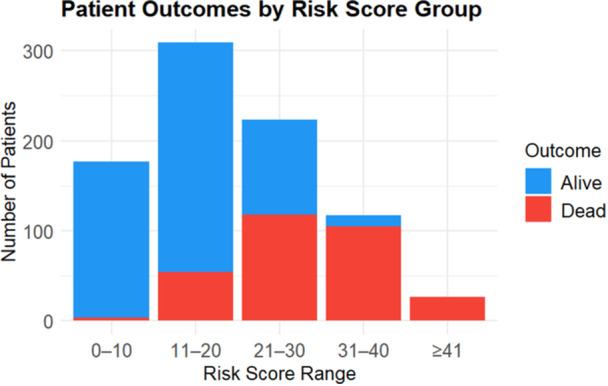
Stacked bar chart showing actual patient outcome by risk stratum.

For clinical use, a simplified risk score was developed from the validated model. The maximum total risk score was 52. For ease of interpretation in clinical settings, the score was dichotomized using the optimal cut‐off points determined by the Youden index, which identifies the value that maximizes the sum of sensitivity and specificity. This method provides an optimal balance between correctly identifying patients at high risk of mortality and minimizing false positives. A score of 21 points corresponded to a predicted probability of 0.6. Accordingly, patients who scored < 21 points were categorized as low risk for ICU mortality, and patients scoring 21 or more were classified as high risk for ICU mortality. The stacked bar chart in Figure 4 also shows that the probability of death increases when the risk stratum increases.

For more simplification, a nomogram was developed based on the simplified risk score model. The nomogram allows for quick calculation of an individual patient's risk score. Clinicians can estimate the score by identifying the patient's values for each predictor on the left side of the nomogram, then drawing a vertical line upward to determine the corresponding score at the top of the chart. The individual scores are then summed to obtain a total risk score, which can be compared against the risk scale provided at the bottom of the nomogram (Table 4).

**Table 4:** Nomogram for predicting the probability of mortality among patients admitted to the ICU in Bahir Dar city referral hospitals, 2019–2021.

Variable	Score								
	0	1	2	3	4	5	6	7	8
Age	15–24	25–34	35–44	45–54	55–64	65–74	≥ 75		
Sex	Female		Male						
Health insurance	Yes			No					
RR (breaths/min)	≤ 20		21–30		31–40		41–50		
Temperature (°C)	≥ 36.1			34.1–36		32–34			
MAP (mmHg)	≥ 70	60–69.99				< 60			
GCS	13–15				9–12		≤ 8		
WBC (1000/mm^3^)	≤ 12		12.1–20		≥ 20.1				
Sepsis	No			Yes					
Acute respiratory distress						Yes			
Organ insufficiency						Yes			
Mechanical ventilator					Yes				
Vasopressor									Yes
Total score	< 21 points (low mortality risk in ICU)				≥ 21 points (high mortality risk in ICU)				

## 4 Discussion

Overall, in this study, 13 variables were identified as independent prognostic determinants for mortality in patients admitted to ICU. These variables were age, health insurance user status, ICU stays, respiratory rate, temperature, mean arterial pressure, Glasgow Coma Scale, WBC, sepsis, ARDS, organ‐insufficiency, mechanical ventilation, and vasopressor.

These variables demonstrated a reasonably predictive ability of ICU mortality. The model developed using these variables produced a discrimination performance of AUC 0.90 (95% CI 0.88–0.92) with a calibration *p* value of 0.69 and calibration curve slope almost overlapped in 45°. This indicates excellent agreement between the predicted and the observed probabilities. The internal validation, which utilized a bootstrap technique, produced a corrected AUC of 0.88, revealing an excellent model performance.

The model developed in this study showed a good ability to discriminate between survivors and nonsurvivors, as evidenced by AUC, or C‐statistics of 0.90. The model was able to assign a higher probability of death to nonsurvivors than to survivors and gave a probability of death for 88% of the dead individuals. This finding is supported by the rule of thumb that models with AUC of 0.8–0.9 are classified as good discriminators [26]. The overall accuracy of the model, given the threshold level of 0.41, was 83.5% (95% CI: 80.9%–86.0%). This means that the model correctly classified 83.5% of all cases into survivors (true negative) and nonsurvivors (true positive). Specifically, 77.8% of the dead patients were correctly classified as dead (sensitivity), and 86.8% of the survivors were correctly classified as survived (specificity).

Based on the identified predictors, a simplified risk score and nomogram were constructed. The simplified risk score had a parallel probability of 0.41 to a score of 21 or more points. Accordingly, patients who scored 21 or more points in the simplified risk score had a higher probability of death. In this sense, the risk score model had an accuracy of 80%, indicating that the misclassification rate for the risk score model was about 20%.

In terms of the AUC or C‐statistics, the risk score performed almost similarly to the original model, with an AUC of 0.89 compared to 0.90 for the original model. Thus, given its simplicity and acceptable performance, the risk score could assist decision‐making in the ICU. This is supported by our DCA, in which the model developed in this study was found to be better than the “no patient treating model” and “the entire patient treating model” (Figure 3).

Our model's performance is comparable to other widely used but more complicated and less affordable clinical prediction systems. For instance, SAPS II demonstrated an AUC of 0.88 in the development sample and 0.86 in the validation sample [13]. Similarly, APACHE IV achieved an AUC of 0.89, MPM0 III showed an AUC of 0.81, and SAPS II resulted in an AUC of 0.87 [27]. In another large validation cohort and a single‐center US study involving 2596 patients, the reported AUCs were as follows: APACHE IV (AUC 0.88 and 0.86), SAPS 3 (AUC 0.85 and 0.80), and MPM0‐III (AUC 0.82 and 0.72), respectively [28].

These findings suggest that our model provides a level of predictive accuracy similar to established scoring systems while potentially offering greater practicality and cost‐effectiveness for routine clinical use. Nevertheless, these comparisons should be interpreted with caution because the previous models were developed and externally validated using substantially larger and more heterogeneous patient populations.

Consistent with our finding, previous studies conducted in low‐income settings have demonstrated that accurate predictive models can be developed using variables that are simple, readily available, and easily measurable [17, 21].

On the other hand, our model's performance was better than that of a model developed in Rwanda, where they found a clinical prediction model with an area under the ROC curve of 0.81 with a 95% CI of (0.77, 0.86), and Hosmer–Lemeshow *χ* ^2^ statistic *p* = 0.15 [17]. This discrepancy may be due to variations in the number of predictors as compared to ours.

In our current data set, we observed a decline in the performance of the Rwanda model, with the AUC decreasing to 78 (95% CI: 74.8–81.2) and accuracy to 73 (95% CI: 74.8–81.2) [17]. We also assessed the Uganda vitals model using our data. In this model, the authors developed a prognostic index using respiratory rates, pulse rates, mean arterial pressures, temperatures and presence of altered mental status. They reported that patients with a score ≥ 3 had a 3.4‐fold higher risk of in‐hospital mortality compared to those with a score < 3 [19]. Using the same five vital signs, we found that patients with a score ≥ 3 were 3.6 times more likely to die in the ICU as compared to those with lower scores.

Overall, as is illustrated in Table 5, the current model demonstrates satisfactory performance, and the variables are easily measurable. These variables could be integrated into an electronic medical record system, allowing for automated calculations and straightforward patient risk classification. Alternatively, the nomogram presented in Table 4 could be utilized for risk estimation, accompanied by a brief explanatory guide.

**Table 5:** Comparison and performance metrics of the current mortality risk score model in Bahir Dar city referral hospitals, 2019–2021.

Model	AUC (development/validation)	Calibration (*p* value/slope)	Setting/notes
Our Model	0.90 (95% CI: 0.88–0.92)	*p* = 0.69; calibration curve≈45° slope	Easily implementable; uses accessible variables; suitable for low‐resource settings
SAPS II [13, 27]	0.88 (dev)/0.86 (val); also 0.873	Not reported	More complex and less affordable; widely used in high‐resource settings
APACHE IV [27, 28]	0.892; also 0.88 (val)/0.86 (single center)	Not reported	High performance; resource‐intensive
MPM0 III [27, 28]	0.8109; also 0.82/0.72	Not reported	Moderate performance; simpler than APACHE but still complex
SAPS 3 [28]	0.85/0.80	Not reported	Good performance in validation but not always consistent
Rwanda Model [17]	0.81 (95% CI: 0.77–0.86)	*p* = 0.154	Lower performance; limited predictors
Rwanda Model (retested)	0.78 (95% CI: 74.8–81.2)	Accuracy: 73%	Performance declined on current data set
Uganda Vitals Model [19]	Not reported	Risk ratio for ≥ 3 score: 3.4× higher mortality	Based on five vital signs (RR, PR, MAP, Temp, AMS)
Uganda Vitals (retested)	Not reported	Risk ratio: 3.6× higher mortality	Consistent results when applied to current data using same five parameters

Abbreviations: AMS = altered mental status, AUC = area under the receiver operating characteristic curve, CI = confidence interval, dev/val = development/validation samples, MAP = mean arterial pressure, PR = pulse rate, RR = respiratory rate, Temp = temperature.

### 4.1 Limitation

This study was retrospective which is inherently susceptible to biases related to data collection and documentation. Additionally, there were instances of missing data, which may have affected model training and performance. Although efforts were made to handle these appropriately, missingness may introduce bias or reduce the robustness of the findings.

Another important limitation concerns the generalizability of our model. The all‐cause mortality risk score was developed using a heterogeneous ICU population, aiming for broad applicability. However, when applied to more specific ICU subgroups, the model's predictive performance may vary. As a result, while the model may perform well on average, its accuracy and utility in specialized ICU contexts require further validation.

## 5 Conclusion and Recommendations

The risk score model developed in this study can be easily used by clinicians, as it relies on routinely collected parameters. Its simplicity makes it suitable for integration into daily decision‐making processes, especially in resource‐limited settings. We recommend that clinicians in the study area consider using this tool to identify high‐risk patients.

However, it is important to exercise caution when applying the model to specific patient populations, as its predictive performance may vary depending on individual patient characteristics and underlying conditions. Importantly, before broader clinical implementation, we strongly recommend prospective validation of the risk score in diverse ICU populations to confirm its reliability, accuracy, and generalizability.

## Author Contributions

Yihun Miskir Wubie conceptualized and designed the study, performed data analysis, interpreted the findings, and drafted the manuscript. Zelalem Alamrew Anteneh supervised every component of the data collection process, performed data analysis, and revised the manuscript. Asiya Mohammed Abdu performed data analysis and critically reviewed the manuscript. All authors read and approved the final version of the manuscript.

## Funding

The authors have nothing to report.

## Ethics Statement

Approval was obtained from the Ethical Review Committee of the College of Medical and Health Science, Bahir Dar University with a Protocol Number 309/2021.

## Consent

Informed consent was not taken as it was not applicable for a retrospective study, and this was also approved by the committee.

## Conflicts of Interest

The authors declare no conflicts of interest.

## Transparency Statement

The lead author Yihun Miskir Wubie affirms that this manuscript is an honest, accurate, and transparent account of the study being reported; that no important aspects of the study have been omitted; and that any discrepancies from the study as planned (and, if relevant, registered) have been explained.

## Data Availability

The data sets generated and/or analyzed during the current study are available from the corresponding author on reasonable request.
